# Aetiology of Community-Acquired Pneumonia and the Role of Genetic Host Factors in Hospitalized Patients in Cyprus

**DOI:** 10.3390/microorganisms11082051

**Published:** 2023-08-10

**Authors:** Petros Ladas, Ilias Porfyridis, Christina Tryfonos, Anna Ioannou, Tonia Adamide, Christina Christodoulou, Jan Richter

**Affiliations:** 1 Molecular Virology Department, Cyprus Institute of Neurology and Genetics, Iroon Avenue 6, 2371 Egkomi, Nicosia, Cyprus; petroslad@gmail.com (P.L.); tryfonos@cing.ac.cy (C.T.); cchristo@cing.ac.cy (C.C.); 2 Pulmonary Department, Nicosia General Hospital, Lemesou 215, 2029 Strovolos, Nicosia, Cyprus; iliasporfi@gmail.com (I.P.); annaioannou_17@hotmail.com (A.I.); tadamide@yahoo.gr (T.A.)

**Keywords:** microorganism detection and analysis, clinical microbiology, community-acquired pneumonia, genomics, real-time PCR/RT-PCR, next-generation sequencing

## Abstract

Community-acquired pneumonia (CAP) remains the leading cause of hospitalization among infectious disease in Europe, and a major cause of morbidity and mortality. In order to determine and characterize the aetiology of CAP in hospitalized adults in Cyprus, respiratory and blood samples were obtained from hospitalized patients with CAP, and analyzed using Multiplex Real-Time PCR/RT-PCR, and ID/AMR enrichment panel (RPIP) analysis. Probe-based allelic discrimination was used to investigate genetic host factors in patients. The aetiology could be established in 87% of patients. The most prevalent viral pathogens detected were influenza A, SARS-CoV-2, and human rhinovirus. The most common bacterial pathogens detected were *Streptococcus pneumoniae*, *Staphylococcus aureus*, and *Haemophilus influenzae*. Antimicrobial resistance genes were identified in 23 patients. *S. aureus* was the most common AMR correlated strain in our study. A positive correlation was detected between bacterial infections and the NOS3 rs1799983 G allele and the FCGR2A rs1801274 G allele. A positive correlation was also detected between the TNF-α rs1800629 A allele and sepsis, while a negative correlation was detected with the ACE rs1799752 insertion genotype and the severity of pneumonia. In conclusion, the targeted NGS panel approach applied provides highly sensitive, comprehensive pathogen detection, in combination with antimicrobial resistance AMR insights that can guide treatment choices. In addition, several host factors have been identified that impact the disease progression and outcome.

## 1. Introduction

Community-acquired pneumonia (CAP) is defined as an acute infection of the pulmonary parenchyma, not acquired in a hospital or a nursing home residence. Common symptoms of CAP are dyspnea, fever, a productive cough, a rapid heartbeat, chest pain, and difficulty breathing, as well as fluid build-up in the lungs [1]. CAP is diagnosed using evidence of the accumulation of fluids in the lung (pulmonary consolidation), along with evidence of an infection [2]. Severe CAP can lead to life-threatening conditions, such as sepsis and acute respiratory distress syndrome (ARDS) [3]. CAP is the leading cause of sepsis, with 40–50% of septic patients being identified as having a respiratory infection as the source [4].

The rapid diagnosis of the etiologic agent in CAP is crucial for the appropriate management of the disease, and for guiding the rational use of antivirals and antibiotics. Published data on the etiology and frequency of CAP causative agents among the Cypriot population do not exist. Therefore, we designed a prospective observational study in collaboration with Nicosia General Hospital, which aimed to determine and characterize, for the first time, the aetiology of CAP in hospitalized adults in Cyprus. In addition, the identified bacteria were analyzed with regard to their resistance profile. Lastly, we aimed to assess genetic host factors associated with CAP severity or progression.

## 2. Materials and Methods

### 2.1. Patients and Study Design

In this observation trial, all consecutive admissions to the Pulmonary Department of Nicosia General Hospital on predetermined and randomly selected emergency duty days were eligible. This study was performed in line with the principles of the Declaration of Helsinki. Approval was granted by the Cyprus National Bioethics Committee (ΕΕΒΚ/ΕΠ/2017/45). Accordingly, written informed consent was obtained from the patients prior to the sample-taking.

The inclusion criteria were: (i) aged above 18 years, (ii) written informed consent; (iii) acute respiratory illness, and (iii) the presence of new pulmonary infiltrates or a pleural effusion on a chest X-ray or on computed tomography of the thorax. Additionally, signs and symptoms of pneumonia, such as (1) a temperature alteration < 36 °C or >38.3 °C, (2) a white blood cell count <4000 cells/mm^3^ or >10,000 cells/mm^3^, (3) an altered mental status, (4) an increase in respiratory secretions or purulent sputum, (5) a new onset of cough or dyspnea, and (6) the presence of rales or bronchial breath sounds, suggested that the pulmonary infiltrates were infectious in origin, and the diagnosis of CAP was established [2]. Pneumonia was considered to be absent when: (i) an alternative cause for the pulmonary infiltrate was established (e.g., a pulmonary embolus), and (ii) full recovery was achieved without antimicrobial therapy.

The exclusion criteria were: (i) human immunodeficiency virus (HIV) infection, (ii) a documented extrapulmonary infection, (iii) recent hospitalization (<60 days), and (iv) a clear alternative diagnosis. Patients were also excluded if they had a tracheotomy, or cystic fibrosis, if they had received a solid-organ or hematopoietic stem-cell transplant within the previous 90 days, or if they had active graft-versus-host disease or bronchiolitis obliterans.

Severe CAP, sepsis, severe sepsis, and septic shock were defined according to current recommendations [5].

Clinical laboratory, and imaging data were recorded for each patient, including: (i) clinical presentation, (ii) body temperature, (iii) arterial blood gas, (iv) peripheral blood cell counts, C-reactive protein and procalcitonin levels, (v) imaging findings, and (vi) in-hospital mortality.

To protect patients’ personal data, each sample was assigned a traceable code number. The respiratory samples obtained included sputum samples, pleural fluid (PF), bronchoalveolar lavage (BAL) samples, when bronchoscopy was performed, and nasopharyngeal samples. Along with the respiratory samples, a peripheral blood sample was also obtained from each patient.

### 2.2. DNA/RNA Extraction from Respiratory Samples

Nucleic acid extraction from the respiratory samples was performed using the MagMax Total Nucleic Acid Isolation Kit (ThermoFisher Scientific, Waltham, MA, USA). The extraction was performed according to the manufacturer’s protocol.

### 2.3. DNA Extraction from Blood

The extraction of DNA from blood samples was performed using the QIAmp DNA Blood Mini kit (QIAGEN, Germany). The extraction was performed according to the manufacturer’s instructions, following the recommended steps for extracting DNA from the buffy coat.

### 2.4. Multiplex Real-Time PCR/RT-PCR for Pathogen Identification

Bacterial pathogen identification assays were carried out in a 25 μL total reaction, with 12.5 μL of TaqMan 2x Universal PCR Mastermix (ThermoFisher Scientific, Waltham, MA, USA), 1 μL of assays primer/probe mix, 6.5 μL ddH_2_O, and 5 μL of extracted DNA; the cycling conditions were as follows: 2 min 50 °C, 10 min 95 °C, followed by 45 cycles of 15 s at 95 °C, and 1 min at 60 °C.

Viral pathogen identification assays were carried out using the AgPath-ID One-Step RT-PCR Kit (Thermo Scientific, Waltham, MA, USA) in a 25 μL total reaction with 12.5 μL of 2x RT-PCR Buffer, 0.9 μL of 25x RT-PCR Enzyme mix, 1 μL of primer/probe mix (containing all the primers and probes for multiplexing), 5.6 μL of ddH_2_O and 5 μL of extracted DNA/RNA; the cycling conditions were as follows: an initial RT step of 30 min at 48 °C, 10 min at 95 °C, followed by 45 cycles of 15 s at 95 °C, and 1 min at 60 °C.

The primers and probes are provided in Supplementary Material (Tables S1 and S2).

### 2.5. Probe-Based Allelic Discrimination

Allelic discrimination assays were carried out using 25 μL total reaction, with 12.5 μL of TaqMan 2x Universal PCR Mastermix (ThermoFisher Scientific, Waltham, MA, USA), 1.25 μL primer/probe mix, and 11.25 μL of extracted DNA; the conditions were as follows: 30 s at 60 °C, 10 min at 95 °C, followed by 45 cycles of 15 s at 95 °C, and 1 min at 60 °C, with a final extension step after the last cycle, for 30 s at 60 °C.

For IL-6 (rs 1800795), IL-10 (rs1800896), ACE (rs1799752,) NOS3 (rs1799980), TNF-α (rs1800629), CYP1A1 (rs2606345), and FCGR2A (rs1801274), polymorphism primers, and probes for allelic discrimination assays were ordered directly from ThermoFisher Scientific. For FUT2 (rs601338), primers and probes were designed in-house, using Primer3Plus, with probes containing zip nucleic acid (ZNA) modifications.

F: GAGGAATACCGCCACATCC, R: GGTCGTGCAGGGTGAACT, P1: HEX- CTGCTCCTGGACCTTCT-ZNA, P2: FAM -CCTGCTCCTAGACCTTCT-.ZNA.

### 2.6. Next-Generation Sequencing

Sequencing libraries were created using the Illumina RNA Prep, Tagmentation (L) with Enrichment (Illumina, Cambridge, UK). The enrichment of libraries was performed using the Respiratory Pathogen ID/AMR Oligo Panel (Illumina, UK). The full protocol that was used in the study is provided as a reference [6]. The validation of the generated libraries was performed using the TapeStation System D1000 (Agilent Technologies, Waldbronn, Germany), and library normalization and pooling was conducted using the Qubit fluorometric quantification (ThermoFisher Scientific, Waltham, MA, USA). The sequencing of libraries was performed via a NexSeq550 Sequencing System (Illumina, Cambridge, UK), using the NextSeq 500/550 High Output kit v2 (75 Cycles) (Illumina, Cambridge, UK). Analysis of the sequencing data was performed using the Explify RPIP Data Analysis software (v2.0.0) available on the BaseSpace platform (Illumina, Cambridge, UK).

### 2.7. Statistical Analysis

Statistical analyses were performed using the RStudio software (Studio Team 2020. RStudio: Integrated Development for R. RStudio, PBC, Boston, MA, USA, URL http://www.rstudio.com/ accessed on 8 June 2022), and the GraphPad Prism software (v9.3.1, Windows, GraphPad Software, San Diego, CA, USA, www.graphpad.com accessed on 16 December 2021). A correlation matrix was created using Spearman’s correlation with GraphPad Prism, to investigate potential associations with the incidence of viral and bacterial co-infections for specific pathogens. Further analysis was performed using binomial logistic regression, using RStudio software. Correlations for the genetic host factors and CAP patients were evaluated using a binomial logistic regression, using RStudio, for the incidence of bacterial and viral infections, as well as sepsis and ARDS in patients. A multinomial logistic regression model was used to analyse the CURB-65 scores and patients’ days of hospitalization with SNP genotypes.

## 3. Results

### 3.1. Patients

**Figure 1:**
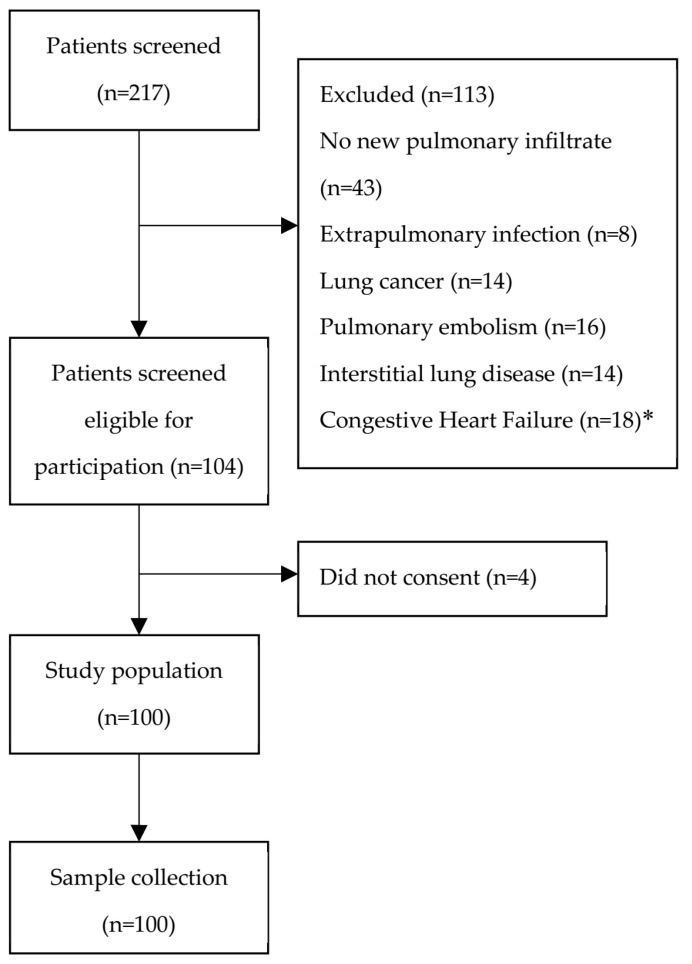
Study flowchart. * Patients with a past medical history of congestive heart failure (CHF) and pneumonia were included; in patients with pulmonary oedema and CHF, without signs of infection, the pulmonary infiltrates were considered noninfectious, and were excluded.

Of the 217 screened patients, 113 were excluded, while four patients did not consent to participate in the study (Figure 1). Patient data were obtained from a total of one hundred patients hospitalized due to CAP. The demographic and clinical data of the patients are summarized in Table 1. Older age in patients was associated with sepsis (*p* = 0.029, r = 0.26) and ARDS (*p* < 0.001, r = 0.46). The average stay at the hospital for patients was eight days. During hospitalization, 20% of these patients were admitted to the intensive care unit (ICU), with 85% of these patients being SARS-CoV-2 patients during the course of the COVID-19 pandemic. The overall 30-day mortality of the patients was 5%. Most of the admitted patients (76.2%) had some form of underlying comorbid condition, with chronic obstructive pulmonary disease (COPD) and congestive heart failure (CHF) being the most common among the admitted patients. A total of 41% (n = 34) of the admitted patients progressed to mild severity ARDS and, in 36% (n = 30) of all patients, pneumonia progressed to sepsis. Of the patients that were identified with ARDS, 61% (n = 21) were also septic.

### 3.2. Characteristics and Microbial Aetiology of CAP

Respiratory pathogens were identified in hospitalized patients through a combination of methods, employing a range of real-time multiplex RT-PCR assays, as well as next-generation sequencing, with RPIP analysis.

Overall, a respiratory pathogen was identified in 87% (n = 87) of patients. Undetected pathogens (13%) were reported when no pathogen could be detected by either real-time PCR or RPIP analysis. Patients were hospitalized due to bacterial infections in 19% (n = 19) of cases, of which 2% (n = 2) were due to infection by two or more bacterial pathogens. In 25% (n = 25) of cases, only viral pathogens were identified, of which 3% (n = 3) were due to infections by multiple viral pathogens. Viral–bacterial co-infections were identified in 43% (n = 43) of hospitalized patients. In 11% (n = 11) of cases, one or more pathogens were identified, alongside a viral–bacterial co-infection.

In Table 2, the identified respiratory pathogens are shown according to the sample type. The detection of a viral pathogen in any of the sample types was considered indicative of an active infection. The detection of bacterial pathogens in the BAL and PF samples was considered indicative of an active infection, due to the presence of the pathogen in the lower respiratory tract. Concerning the sputum and swab samples, the detection of a bacterial pathogen represents the presence of the pathogen, but we cannot distinguish between an active infection and colonization. With regard to methodology, a total of 136 microbial pathogens were detected using real-time PCR, while 96 microbial pathogens were identified via the NGS approach. Of the 96 microbial pathogens detected using the RPIP analysis, three were identified as new pathogens not part of the real-time PCR assays: *Bacteroides fragilis*, *Chlamydia psitacci*, and Herpes simplex virus 1. An additional seven pathogens were identified via the RPIP analysis that were a part of, but not identified by, real-time PCR analysis, while the rest of the verified results were obtained via real-time PCR (Table 3).

**Figure 2:**
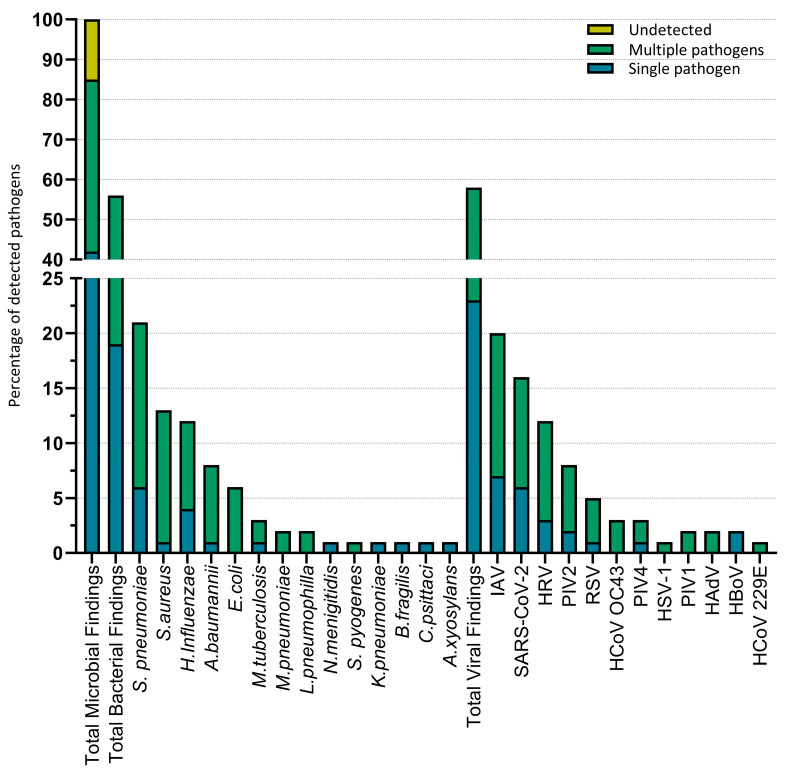
Microbial pathogens detected in CAP patients. Microbial findings in 100 cases, with the proportion of co-infections. Single pathogen refers to cases where only one pathogen was identified in a CAP patient. Multiple pathogens refer to cases where multiple pathogens, viral–bacterial co-infection, multiple bacterial infections, or multiple viral infections were identified in a CAP patient.

The most common bacterial pathogens identified in patients were *S. pneumoniae* in 21% (n = 21), *S. aureus* in 13% (n = 13), and *H. influenzae* in 12% (n = 12), while the most common viral pathogens detected were influenza A virus (IAV) in 20.2% (n = 20), SARS-CoV-2 in 16% (n = 16), and human rhinovirus (HRV) in 12% (n = 12) (Figure 2).

In terms of seasonal viruses, we observed that IAV was predominantly identified during the winter months; specifically, H1N1 IAV was detected from January until March 2019, followed by H3N2 IAV between January and February 2020. Similarly to IAV, HRV infections were identified predominantly in the winter months, with most cases not coinciding with seasonal influenza.

Following the onset of the COVID-19 pandemic, SARS-CoV-2 became the most prevalent pathogen among CAP patients in Cyprus. We report that SARS-CoV-2 was identified in 16% of patients, all of whom had been hospitalized in the intensive care unit.

The pathogens detected in CAP patients were further analyzed in terms of their rate of co-infection, and the potential significant correlation between pathogens. Of the commonly detected pathogens, *S. aureus* had the highest rate of both viral/bacterial and bacterial/bacterial co-infection at 92.3% (n = 12), followed by *S. pneumoniae* at 71.4% (n = 15), and *H. influenzae* at 66.6% (n = 8). For viral pathogens HRV had the highest rate of co-infection at 66.7% (n = 8), followed by SARS-CoV-2 at 56.2% (n = 9), and IAV at 55% (n = 11). The pathogens identified only in viral–bacterial co-infections in more than one patient were *E. coli* (n = 6), HCoV-OC43 (n = 3), M. pneumoniae (n = 2), PIV-1 (n = 2), and HAdV (n = 2). *A. baumannii* had the highest viral/bacterial co-infection rate among the bacterial pathogens, at 87.5% (n = 7), with a strong correlation showing between SARS-CoV-2 and *A. baumannii* (β = 2.6, OR = 13.8, *p* = 0.004). The correlations between co-pathogens were analyzed firstly using Spearman’s correlation for the incidence of infections across all samples, followed by logistic regression. We identified a strong correlation between *S. aureus* and PIV-2 (β = 2.4, OR = 11.7, *p* = 0.027), and between *S. aureus* and *E. coli* infections (β = 4.37, OR = 79, *p* < 0.001). Lastly, we identified a strong correlation between IAV H3N2 infection and *S. pneumoniae* (β = 2.6, OR = 13.4, *p* = 0.027).

### 3.3. Antimicrobial Resistance

Respiratory pathogens were analyzed in terms of antimicrobial resistance (AMR), using next generation sequencing, employing the Respiratory Pathogen ID/AMR Enrichment Panel kit (RPIP). The resulting data were analyzed using the Explify RPIP Data analysis software (v2.0.0). In terms of antimicrobial resistance genes, results correlating with commensal bacteria for which no literature existed supporting respiratory infections were excluded. Of a total of 58 samples that were identified as positive for bacterial infections, 23 (39.6%) indicated bacterial infections associated with AMR genes. A total of 67 AMR genes were identified across all samples. Antimicrobial resistant *S. aureus* was the most common AMR-correlated strain in our study (n = 8). In four samples, *S. aureus* was identified as methicillin-resistant *S. aureus* (MRSA), due to the presence of the mecA gene. The rest of the *S. aureus* cases (n = 4) were correlated with macrolide and lincosamide resistance, due to the presence of the Erm and ABC-F genes (Table 4).

The RPIP analysis also allowed the characterization of the identified IAV. Of the 13 IAV cases identified using the NGS approach, six were classified as H1N1, and seven as H3N2. All the H3N2 was identified as neuraminidase-inhibitor-resistant, due to mutations in the neuraminidase (NA) gene. In five cases, the S245N mutation of NA was identified, while, in two cases, the N294S mutation was detected.

Each sample was analyzed for potential antimicrobial resistance to drug classes associated with the AMR genes discovered. The most prevalent antimicrobial resistance to drug classes identified in our samples were to macrolides (18.7%), followed by aminoglycosides (16.2%), and penicillin (15%).

### 3.4. Host Factors Associated with CAP

**Figure 3:**
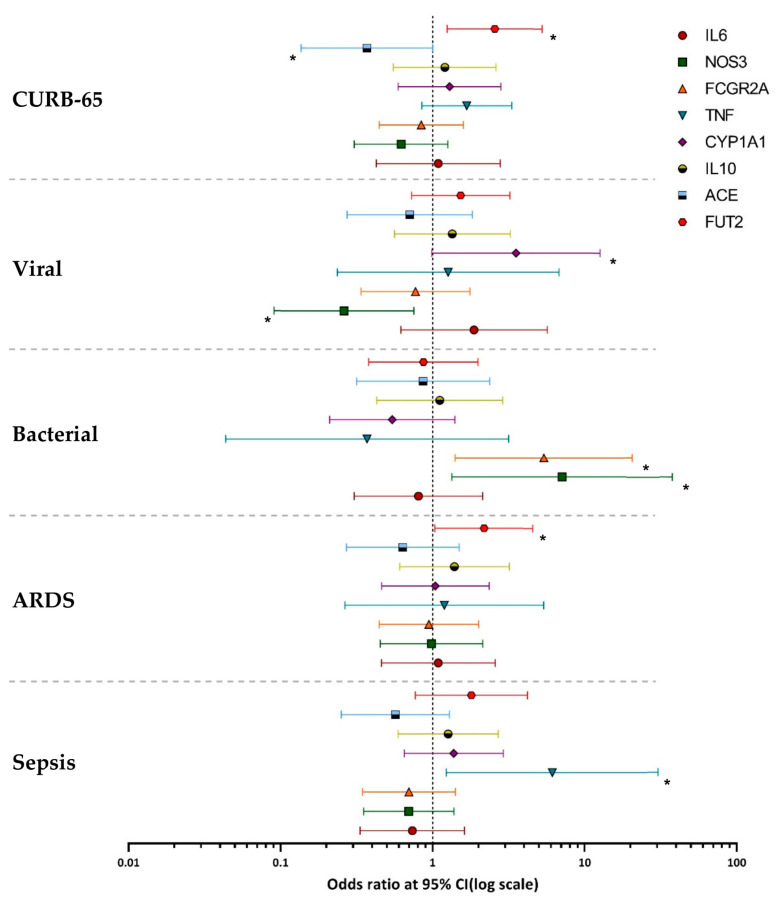
Association of genetic host factors with CAP severity and disease. Logarithmic scale of odds ratios of genetic host factors, with the error bars representing confidence intervals of 95%. Viral infection represents the incidence of single viral infections in the patient population. Bacterial infection represents the incidence of single bacterial infections in the patient population. ARDS represents the number of patients with acute respiratory distress syndrome. Sepsis represents the number of patients diagnosed as septic during hospitalization. * Indicates that the logistic regression was statistically significant (*p* value ≤ 0.05).

Following the probe-based allelic discrimination assay, the genotypes of each sample were recorded, and categorized according to each SNP (Table 5). A binary logistic regression model was used to correlate the occurrence of bacterial infections with each SNP. In this model, each SNP was analyzed for the gain of mutant alleles, and the incidence of viral or bacterial infections, against the homozygote wild-type genotype of each SNP. The NOS3 (rs1799983) G allele and the FCGR2A (rs1801274) G allele were positively correlated with the occurrence of bacterial infections in patients; β = 1.96, OR = 7.12, *p* value = 0.0212; and β = 1.68, OR = 5.4, *p* value= 0.014, respectively. When analyzed with respect to the occurrence of viral infections, a positive correlation was observed with the CYP1A1 (rs2606345) A allele (β = 1,26 OR = 3.53 *p* value = 0.05). When the SNP genotypes were analyzed with respect to the occurrence of co-infections in the samples, no significant correlation was observed. No correlation was identified between the incidence of bacterial or viral infections with the SNPs rs1800795 for IL6, rs1800896 for IL10, rs601338 (FUT2), rs1800629 (TNF-α), and rs1799752 (ACE) (Figure 3).

The genotype profile of each sample was then analyzed with regard to the severity of CAP in each patient. The TNF (rs1800629) A allele was positively correlated with sepsis in CAP patients, with β = 1.81, OR = 6.14, *p* value= 0.027. The FUT2 (rs601338) A allele was positively correlated with ARDS in patients with β = 0.78, OR = 2.18, *p* value= 0.039. A positive correlation with the CURB-65 scores was observed for the FUT2 (rs601338) A/A genotype (β = 0.94, OR = 2.56, *p* value = 0.01), while a negative correlation with the CURB-65 scores was observed for the ACE (rs1799752) ins/ins genotype (β = −0.99, OR = 0.37, *p* value = 0.05). No correlation was identified between CAP sepsis or ARDS or CURB-65 scores with the SNPs rs1800795 (IL6), rs1800896 (IL10), rs1799983 (NOS3), rs1801274 (FCGR2A), and rs2606345 (CYP1A1) (Figure 3).

No significant correlation was observed for the SNPs rs1800795 (IL-6) and rs1800896 (IL-10) when analyzed with regard to the incidence of viral or bacterial infections, or when analyzed in respect of CAP severity.

## 4. Discussion

### 4.1. Hospitalized Patient Characteristics

The data collected during the admission of patients highlight the significance of the risk factors associated with CAP [7]. Underlying comorbid conditions are crucial risk factors associated with CAP. We observed that 76% of patients had some form of underlying comorbid condition, with lung disorders (COPD and asthma) and CHF being the most common among our population. In our study, the median age of patients was 62.5 years old, lower than what is considered a risk factor for CAP (over 65 years), although older patients were associated with poor clinical outcomes (sepsis and ARDS). The shift of the median age of patients to below 65 can be attributed to lifestyle factors, such as smoking: as of 2018, Cyprus is ranked as the European country with the sixth-highest number of tobacco smokers [8].

### 4.2. Aetiology of CAP and Antimicrobial Resistance in CAP Patients

This three-year prospective study is the first study conducted in Cyprus to characterize the aetiology of CAP in hospitalized adults. The aetiology of CAP was established in the majority of patients, with *S. pneumoniae* and influenza A (IAV) being the most commonly detected respiratory pathogens in patients. Our results are in accordance with other similar studies that report *S. pneumoniae* and IAV as the most prevalent pathogens in CAP patients [9]. In terms of viral pathogens, the most prevalent in our patient population were IAV, SARS-CoV-2, and human rhinovirus (HRV). Regarding bacterial pathogens, the most prevalent were *S. pneumoniae*, *S. aureus*, and *H. influenzae*. In 5% of patients, a bacterial pathogen commonly associated with atypical pneumoniae was identified; this would be one out of *M. pneumoniae*, *L. pneumophila*, *C. psittaci*, and *M. tuberculosis* [10]. Interestingly, we identified only one case of *K. pneumoniae*, a bacterium that, in similar studies, was identified as a common pathogen in CAP patients [11]

The overwhelming majority of *A. baumannii* detected was from ICU patients during the COVID-19 pandemic. The bacterium was identified primarily alongside SARS-CoV-2, and was characterized by multiple resistance genes; most notably, the oxacillinase OXA-23, a β-lactamase that confers carbapenem resistance [12]. The prevalence of carbapenem-resistant *A. baumannii* (CRAb) strains has been increasing in Europe over recent years, and has been associated with high fatality rates [13]. The WHO has listed CRAb as a priority for the research and development of new antibiotics. Several studies have reported outbreaks of CRAb during the onset of the COVID-19 pandemic, associated with high mortality rates. These outbreaks appear to be associated with the high rate of hospital admissions, the need for the mechanical ventilation of patients due to the severe pulmonary symptoms caused by SARS-CoV-2, and poor infection-control measures [14,15].

In terms of antimicrobial resistance, *S.aureus* had the highest rate of AMR genes among the pathogens commonly associated with CAP. Half of the AMR-correlated infections were identified as macrolide-resistant, due to the presence of the Erm and ABC-F genes, while four *S. aureus* infections were identified as MRSA, due to the presence of the methicillin-resistant gene mecA [16]. Of significance was the identification of two *E. coli* infections and one *H. influenzae* infection carrying the beta-lactamase TEM-1 AMR gene, which confers the extended spectrum beta lactamase (ESBL) phenotype to the bacterium [17]. In addition, all the infections by H3N2 were characterized by a resistance to neuraminidase inhibitors, due to the neuraminidase (NA) mutations S245N and N294S. The S245N mutation is associated with reduced inhibition by oseltamivir and zanamivir, two commonly used neuraminidase inhibitors, while the N294S confers resistance to oseltamivir [18].

In terms of viral/bacterial co-infections among respiratory pathogens, we observed a significant correlation between the incidence of SARS-CoV-2 and *A. baumannii*, which, as discussed above, was probably due to the high rate of hospital admissions. Another significant correlation was observed between the H3N2 subtype of IAV and *S. pneumoniae*. IAV infections are known to predispose patients to secondary bacterial infections, with *S. pneumoniae* and *S. aureus* being the most common bacterial pathogens detected in such infections [19]. Another correlation identified was between PIV-2 and *S. aureus*. Although parainfluenza viruses have been shown to promote the adhesion of *S. pneumoniae* and *H. influenzae* to respiratory epithelial cells, the synergistic mechanism between PIVs and *S. aureus* remains unknown [20].

### 4.3. Host Factors and the Incidence of Viral and Bacterial Infections

A positive correlation between the incidence of bacterial infection, and the gain of a mutant allele, was observed for the polymorphisms rs1799983 (NOS3) and rs1801274 (FCGR2A). NOS3 is produced by endothelial cells, and is responsible for the synthesis of nitric oxide (NO) [21]. During an infection, endothelial NO (eNO) is produced in far-lower quantities than inducible NO (iNO), with the role of NOS3 appearing to be more regulatory [22]. It has been found that rs1799983 reduces the activity of NOS3, leading to a reduced production of endothelial NO [23]. To our knowledge, there has been no description of the association of rs1799983 with bacterial infections. Given the fact that human airway epithelial cells can only express NOS3 [21], we suspect that the correlation observed in our study was driven by a reduced production of eNO that can affect the regulation of innate immune cells during infection.

FcγRII is a surface receptor found on immune cells, with the variant FcγRIIa (CD32a) mainly expressed on neutrophils, macrophages, and dendritic cells [24]. The CD32a receptor is the only one capable of interacting with IgG2, which is specifically directed against encapsulating bacteria [25]. The rs1801274 polymorphism has been shown to have a reduced affinity to IgG2, with macrophages carrying the variant alleles of the polymorphism having a reduced phagocytosis of IgG2-opsonized particles [26]. Results from the literature vary significantly with regard to the effects of the rs1801274 polymorphism in pneumonia patients [27,28], but there is evidence that the polymorphism is associated with pneumococcal bacteraemia in CAP patients [27]. The majority of detected bacterial infections in our patients were caused by encapsulated bacteria, which supports the hypothesis that the polymorphism results in a reduced clearance of encapsulated bacteria among pneumonia patients.

The CYP1A1 is a member of the cytochrome P450 superfamily, involved in the metabolism of a broad spectrum of xenobiotics and endobiotics [29]. Recent studies have identified CYP1A1 as a regulator of the immune responses affecting the balance of reactive oxygen species (ROS), and the production of TNF-α and IL-6 [29,30]. It has been identified that rs260345 is a functional polymorphism of CYP1A1, with the presence of the variant A allele resulting in a lower gene expression [31]. Interestingly, a recent study reported a positive correlation between the A allele of rs260345 and the prevalence of SARS-CoV-2 in populations worldwide [32]. Although the A allele of the polymorphism has been found to reduce the promoter activity of CYP1A1, a functional correlation between the polymorphism and a predisposition to viral infections requires further investigation.

### 4.4. Genetic Host Factors Associated with CAP Severity

We found that the A allele of the rs1800629 polymorphism of TNF-α was positively correlated with sepsis in our patient population, with the results not being affected by the ARDS or CURB-65 scores. Tumor necrosis factor alpha is a pro-inflammatory cytokine with a diverse range of immunomodulatory effects. Several studies have reported an association between TNF-α and sepsis, reporting that an overexpression of TNF-α leads to poorer outcomes [33]. The rs1800629 variant A allele has been associated with sepsis, and is linked with an elevated gene expression [33]. In our study, the variant A allele was identified in 15.6% of patients.

Gaining the variant A allele of rs601338 was correlated with mild-severity ARDS in our patients, with 16.9% of our patients having the A/A genotype. A recent study by Reily et al. demonstrated the association between the ABO blood group A and ARDS. The association was driven by the A1 subtype of blood group A, and was present in FUT2-determined non-secretors [34]. We believe that the positive association observed in our study between the A/A genotype of rs601338 and increased CURB-65 scores in patients can mainly be attributed to the association of the polymorphism with ARDS. As the calculated CURB-65 scores take into account the respiratory rate of patients, ARDS is correlated with increased CURB-65 scores. Several studies have reported that the phenotypes of FUT2 can confer susceptibility to either bacterial or viral infections, best summarized by Taylor et al. [35]; however, we did not observe any significant association between the rs601338 of FUT2 and the incidence of either bacterial or viral infections. The difference in results can be explained by the fact that several of the studies included the multiple loss of function polymorphisms, including rs601338, to determine the susceptibility to certain pathogens [36].

Lastly, we observed a negative correlation between the rs1799752 I/I genotype of ACE and increased CURB-65 scores in patients. The I/I genotype was identified in 6.7% of patients. Both ACE and angiotensin II have been shown to act as modulators of inflammatory responses [37]. Several studies have reported the protective effects of the I/I genotype in pneumonia patients, which is thought to be related to lower levels of pro-inflammatory angiotensin II, due to the I/D and I/I polymorphisms [38,39]. Additionally, patients with the I/I and I/D genotypes were found to have a better cough reflex than patients with the D/D genotype, due to lower tissue levels of bradykinin [40].

### 4.5. Limitations

For the characterization of genetic host factors associated with CAP, the principal limitation of the study was the exclusion of a control group in the study design. The polymorphisms we chose to investigate in our study had already been associated with CAP in hospitalized patients in other studies [41,42]. The purpose of this study was to investigate the potential effects of the polymorphisms in CAP hospitalized adults; therefore, each SNP was investigated under the premise that it is associated with CAP, and potentially exerts an effect on the progression of the disease. As for the detection of respiratory pathogens, a quantitative approach to real-time PCR would have allowed us to introduce a genome copy cut off for bacterial pathogens, and thus would been more appropriate for differentiation between an active infection and colonization in sputum and nasopharyngeal samples.

## 5. Conclusions

This is the first study in Cyprus to characterize the aetiology of CAP in hospitalized adults. The targeted NGS panel approach applied provides highly sensitive, comprehensive pathogen detection, in combination with antimicrobial resistance AMR insights that can guide treatment choices. The most common pathogens detected were *S. pneumoniae* and influenza A virus. A strong association between H3N2 IAV and *S. pneumoniae* co-infections was observed. With regard to AMR, the most common antimicrobial-resistant pathogen in CAP patients in Cyprus was *S. aureus*, with 50% of identified cases being methicillin-resistant strains. Almost 40% of the bacterial strains identified in our study were shown to possess an antimicrobial resistance, with macrolides being the most common drug class being affected. A genetic profile of the polymorphisms associated with CAP was created for all hospitalized patients, and identified several host factors that impacted the disease progression and outcome. A total of 15% of hospitalized patients with a poor clinical outcome were associated with the rs1800629 polymorphism of TNF-α that is strongly correlated with sepsis, while 6.7% of patients were associated with a less severe form of CAP, due to the rs1799752 I/I genotype of ACE. For the polymorphisms rs1799983 (NOS3), rs1801274 (FCGR2A), and rs260345 (CYP1A1), further investigation is required, to better elucidate their role in CAP. For rs601338 (FUT2), in contrast to other studies, our results did not show a correlation of the SNP with resistance to viral or bacterial infections; however, we observed an indication that the SNP may indirectly affect the progression of ARDS in patients.

## Figures and Tables

**Table 1:** Characteristics of hospitalized CAP patients.

Characteristics	Patients
Age ꝉ	62.5 (18–90) *
Female/Male ꝉ	33/67
Patients with comorbid conditions	64 (76.2)
COPD	19 (22.62)
Asthma	8 (9.52)
Neoplastic disorder	8 (9.52)
Renal disease	9 (10.71)
Congestive heart failure	17 (20.24)
Immunosuppression	6 (7.14)
Diabetes	11 (13.10)
Liver disease	1 (1.19)
Pneumococcal vaccine	5 (5.95)
Influenza vaccine	7 (8.33)
Antibiotics prior to hospital admission	13 (15.48)
CURB-65	2 (0.12) ‡
Days of hospitalization	6 (4–10) §
In-hospital mortality	3 (3.6)
30-day mortality	2 (2.4)
White blood cell count	
4000 cells/mm^3^	0
10,000 cells/mm^3^	46 (54.8)
C-reactive protein > 80 mg/L	49 (58.3)
Pa_O2_/Fi_O2_ ≤ 250 mmHg	17 (20.2)
Chest infiltrates	
Left	26 (31)
Right	30 (35.7)
Bilateral	18 (21.4)
Parapneumonic pleural effusions	11 (13.1)
Empyema	3 (3.6)

Note: Values are presented as No (%), * Median age of patients (range of patients’ age). ꝉ For SARS-CoV-2 patients, the data collected included only age and sex. ‡ Mean CURB-65 scores for all patients (standard error). § Median days of hospitalization of patients (interquartile range).

**Table 2:** Respiratory samples of CAP patients.

	Sputum			BAL			PF			Swab		
	Total	+	%	Total	+	%	Total	+	%	Total	+	%
Total samples	45	41	91.1	33	28	84.8	15	10	66.7	7	6	85.7
Bacterial infection		10	22.2		6	18.2		2	13.3		2	28.6
Viral infection		6	13.3		11	33.3		7	46.7		1	14.3
Viral/bacterial co-infection		25	55.6		11	33.3		1	6.7		3	42.9
Negative		4	8.9		5	15.2		5	33.3		1	14.3

Note: Table describes the total number of each type of respiratory sample: sputum, BAL, PF, and swab, followed by the number of samples positive to different types of infections under column (+). Bacterial infection includes single bacterial and bacterial/bacterial infections; viral infection includes single viral and viral/viral infections, viral/bacterial includes viral/bacterial co-infections. The number of samples in which no pathogen was identified is reported as negative.

**Table 3:** Respiratory pathogens identified in CAP samples.

Pathogens		Sputum (n)	BAL (n)	PF (n)	Swab (n)	Real-Time PCR/RT-PCR	RPIP Analysis	
Total respiratory pathogens		79	46	13	11	136	96	10
Bacterial pathogens		46	18	3	6	69	51	4
*Acinetobacter baumannii*	✓	0	7	0	1	8	6	
*Achromobacter xyosylans*	✓	1	0	0	0	1	0	
*Bacteroides fragilis*		0	1	0	0	0	1	1
*Chlamydia pneumoniae*	✓	0	0	0	0	0	0	
*Chlamydia psittaci*		1	0	0	0	0	1	1
*Escherichia coli*	✓	4	2	0	0	6	2	
*Haemophilus influenzae*	✓	10	1	0	1	12	12	
*Klebsiella pneumoniae*	✓	0	0	0	1	0	1	1
*Legionella pneumophilla*	✓	1	1	0	0	2	2	
*Moraxella catarrhalis*	✓	0	0	0	0	0	0	
*Mycoplasma pneumoniae*	✓	2	0	0	0	2	1	
*Mycobacterium tuberculosis*	✓	2	0	0	1	3	3	
*Neisseria meningitidis*	✓	0	0	1	0	1	0	
*Pseudomonas aeruginosa*	✓	0	0	0	0	0	0	
*Staphylococcus aureus*	✓	9	3	0	1	13	8	
*Streptococcus pneumoniae*	✓	16	3	1	1	20	14	1
*Streptococcus pyogenes*	✓	0	0	1	0	1	0	
Viral pathogens		33	28	9	5	67	45	6
Human adenovirus	✓	2	0	0	0	1	0	
Human bocavirus	✓	0	0	2	0	2	0	
Human coronavirus OC43	✓	3	0	0	0	3	1	
Human coronavirus 229E	✓	1	0	0	0	1	1	
Human coronavirus NL63	✓	0	0	0	0	0	0	
Human coronavirus HKU1	✓	0	0	0	0	0	0	
Human metapneumovirus	✓	0	0	0	0	0	0	
Human rhinovirus	✓	6	1	4	1	10	8	2
Herpes simplex virus 1		0	1	0	0	0	1	
Influenza A	✓	13	5	0	2	17	13	3
Influenza B	✓	0	0	0	0	0	0	
Influenza C	✓	0	0	0	0	0	0	
Human parainfluenza virus 1	✓	1	0	0	1	2	0	
Human parainfluenza virus 2	✓	4	3	1	0	8	2	
Human parainfluenza virus 3	✓	0	0	0	0	0	0	
Human parainfluenza virus 4	✓	0	0	2	1	3	0	
Respiratory syncytial virus	✓	3	2	0	0	5	3	
Severe acute respiratory distress syndrome coronavirus 2	✓	0	16	0	0	15	16	1

Note: Table includes all pathogens that were investigated in the study. ✓ represent pathogens that were included in the multiplex real-time PCR/RT-PCR assays. Column RPIP reports all pathogens identified using the RPIP analysis; the second column under RPIP represents the number of pathogens identified using the RPIP analysis that were not previously identified using real-yime PCR/RT-PCR. Abbreviations: (1) BAL—bronchoalveolar lavage fluid samples, (2) PF—pleural fluid samples, (3) swab—nasopharyngeal swab samples, (4) RPIP—respiratory pathogen ID/AMR enrichment panel.

**Table 4:** Antimicrobial resistance of bacterial pathogens detected in CAP patients.

Pathogens Correlated with AMR	AMR Family	AMR Gene	AMR Identified (n)	Confidence Score	AMR Associated Drug Class	
*Streptococcus pneumoniae*	Tet	tetM	1	High	Tetracycline	
	ABC-F	mel	1	Medium	Lincosamide Oxazolidinone	Macrolide Tetracycline
	Erm	ErmC	1	High	Lincosamide	Macrolide
*Staphylococcus aureus*	Erm	ErmB	1	High	Lincosamide	Macrolide
	Erm	ErmF	1	High	Lincosamide	Macrolide
	ABC-F	mel	1	Medium	Lincosamide Oxazolidinone	Macrolide Tetracycline
	ABC-F	lsaC	2	Medium	Lincosamide Oxazolidinone	Macrolide Tetracycline
	blaZ	blaZ	2	High	Penicillin	
		mecA	4	High	Beta-Lactam Carbapenem Cephalosporins 1–4	Beta-lactamase Inhibitor Penicillin
	LNU	lnuC	1	High	Lincosamide	
	ANT(4’)	ANT(4’)-Ib	2	High	Aminoglycoside	
	Dfr	dfrC	1	High	Diaminopyrimidine	
*Escherichia coli*	Erm	ErmC	1	High	Lincosamide	Macrolide
	Dfr	dfrC	2	High	Diaminopyrimidine	
	ANT(4’)	ANT(4’)-Ib	2	High	Aminoglycoside	
	TEM	TEM-1	1	High	Penicillin	
	Sul	sul2	1	High	Sulfonamide	
	Erm	ErmX	1	High	Lincosamide	Macrolide
*Acinetobacter baumannii*	ABC-F	msrE	6	High	Lincosamide Oxazolidinone	Macrolide Tetracycline
	Sul	Sul1	4	High	Sulfonamide	
	OXA	OXA-23	6	High	Carbapenem Penicillin	Cephalosporin 1st Cephalosporin 3rd
	16S RMTase	armA	6	High	Aminoglycoside	
	MPH	mphE	6	High	Macrolide	
*Mycobacterium tuberculosis*	Qnr	mfpA	2	High	Fluoroquinolone	
	AAC(2’)	AAC(2’)-Ic	2	High	Aminoglycoside	
	Erm	ErmB	1	High	Lincosamide	Macrolide
*Haemophilus influenzae*	TEM	TEM-1	2	High	Penicillin	
*Bacteroides fragilis*	Tet	tetQ	1	High	Tetracycline	
*Klebsiella pneumoniae*	Qnr	QnrD1	1	High	Fluoroquinolone	
	ANT(4’)	ANT(4’)-Ib	1	High	Aminoglycoside	
**Pathogens correlated with AMR variants**	**Gene name**	**Nucleotide change**	**Protein Change**	**Allele frequency**	**Depth**	**Drug Resistance**
*Streptococcus pneumoniae*	parC	245C > T	S82F	1	80	Fluoroquinolone
*Mycobacterium tuberculosis*	embC	2941G > C	V981L	1	26	Polyamine antibiotic
	thyA	604A > G	T202A	1	101	Para-aminosalicylic Acid

Note: Descriptive results for each pathogen correlated with the acquired AMR genes, including the gene family, gene name, confidence scores for the correlation between pathogen and AMR gene as reported by the Explify analysis software, and associated drug class that the AMR genes are resistant to. Reported are single point mutations on bacterial genes that are associated with antimicrobial resistance.

**Table 5:** Frequency of genetic host factors associated with CAP in patients.

SNP	Gene	Homozygote Wild-Type n (%)	Heterozygote n (%)	Homozygote Mutant n (%)
**rs601338**	**Fut2**	G/G n = 35 (42.17)	G/A n = 34 (40.1)	A/A n = 14 (16.9)
**rs1800795**	**IL6**	C/C n = 4 (5.2)	C/G n = 23 (29.9)	G/G n = 50 (64.9)
**rs1799983**	**NOS3**	T/T n = 6 (8.1)	T/G n = 33 (44.6)	G/G n = 35 (47.3)
**rs1801274**	**FCGR2A**	A/A n = 27 (35.5)	A/G n = 38 (50)	G/G n = 11 (14.5)
**rs1800629**	**TNF**	G/G n = 65 (84.4)	G/A n = 12 (15.6)	A/A n = 0
**rs2606345**	**CYP1A1**	C/C n = 10 (13)	C/A n = 40 (52)	A/A n = 27 (35)
**rs1800896**	**IL10**	T/T n = 31 (40.2)	T/C n = 39 (50.7)	C/C n = 7 (9.1)
rs1799752 *	**ACE**	del n = 29 (38.7)	del/ins n = 41 (54.7)	ins n = 5 (6.7)

Note: Frequency of genotypes detected vis probe-based allelic discrimination in hospitalized CAP patients, reported as a number (percentage). * The ACE polymorphism includes the deletion or insertion of the Alu repetitive element in the intron of the ACE gene.

## Data Availability

The data presented in this study are available on request from the corresponding author. The data are not publicly available, due to privacy and ethical restrictions.

## Supplementary Materials

The following supporting information can be downloaded at: https://www.mdpi.com/article/10.3390/microorganisms11082051/s1, Table S1: Primers and probes for identification of bacterial pathogens, Table S2: Primers and probes for identification of viral pathogens. References [43,44,45,46,47,48,49,50,51,52,53,54,55,56,57,58,59] are cited in the supplementary materials.
