# Supplementary material for: Aetiology of Community-Acquired Pneumonia and the Role of Genetic Host Factors in Hospitalized Patients in Cyprus
Source: Microorganisms. 2023 Aug 10;11(8):2051. doi: 10.3390/microorganisms11082051 (PMC10458012; doi:10.3390/microorganisms11082051)
Supplement: Supplementary file 1 [file microorganisms-11-02051-s001.zip › microorganisms-2513947-supplementary.pdf]

Table.S1 Primers and probes for identification of bacterial pathogens

	Gene		Sequence 5′→3′	Probe dye	Concentration [μM]	Reference
Streptococcus pneumoniae	lytA	F	ACGCAATCTAGCAGATGAAGCA		0.25	[43]
		R	TCGTGCGTTTTAATTCCAGCT		0.25	
		P	CCGAAAACGCTTGATACAGGGA-BHQ1	FAM	0.1	
Haemophilus influenzae	fuckP	F	ATGGCGGGAACATCAATGA		0.1	[43]
		R	ACGCATAGGAGGGAAATGGTT		0.3	
		P	CGGTAATTGGGATCCAT-MGB	TAMRA	0.1	
Neisseria meningitidis	crtA	F	GCTGCGGTAGGTGGTTCAA		0.3	[44]
		R	TTGTCGCGGATTTGCAACTA		0.3	
		P	CATTGCCACGTGTCAGCTGCACAT	YY	0.1	
Mycoplasma pneumoniae	CARDS toxin	F	TTTGGTAGCTGGTTACGGGAAT		0.5	[45]
		R	GGTCGGCACGAATTTCATATAAG		0.5	
		P	TGTACCAGAGCACCCCAGAAGGGCT	FAM	0.1	
Chlamydophila pneumoniae	ompA	F	GGGCTATAAAGGCGTTGCTTT		0.5	[46]
		R	AGACTTTGTTCCAGTAGCTGTTGCT		0.5	
		P	CCTTGCCAACAGACGCTGGCG	HEX	0.2	
Klebsiella pneumoniae	gtlA	F	AGGCCGAATATGACGAAT		0.25	[43]
		R	GGTGATCTGCTCATGAA		0.25	
		P	ACTACCGTCACCCGCCACA	TAMRA	0.2	
Staphylococcus aureus	nuc	F	AGCATCCTAAAAAAGGTGTAGAGA		0.4	[43]
		R	CTTCAATTTTMTTTGCATTTTCTACCA		0.4	
		P	TTTTCGTAAATGCACTTGCTTCAGGACCA	FAM	0.1	
Moraxella catarrhalis	copB	F	CGTGTTGACCGTTTTGACTTT		0.15	[43]
		R	CATAGATTAGGTTACCGCTGACG		0.15	
		P	ACCGACATCAACCCAAGCTTTGG	YY	0.1	
Pseudomonas aeruginosa	gyrB	F	CCTGACCATCCGTCGCCACAAC		0.25	[43]
		R	CGCAGCAGGATGCCGACGCC		0.25	
		P	CCGTGGTGGTAGACCTGTTCCCAGACC	TAMRA	0.1	
Escherichia coli	yccT	F	ATCGTGACCACCTTGATT		0.25	[43]
		R	TACCAGAAGATCGACATC		0.25	
		P	CATTATGTTTGCCGGTATCCGTTT	FAM	0.1	

Legionella pneumophila	mip	F	AAAGGCATGCAAGACGCTATG		0.4	[47]
		R	GAAACTTGTTAAGAACGTCTTTCATTTG		0.4	
		P	TGGCGCTCAATTGGCTTTAACCGA	HEX	0.1	
Mycobacterium tuberculosis	IS6110	F	AGACGTTATCCACCATAC		0.4	[48]
		R	AGTGCATTGTCATAGGAG		0.4	
		P	TCTCAGTACACATCGATCCGGT	TAMRA	0.1	
Streptococcus pyogenes	spy	F	GCACTCGCTACTATTTCTTACCTCAA		0.3	[49]
		R	GTCACAATGTCTTGGAAACCAGTAAT		0.3	
		P	CCGCAACTCATCAAGGATTTCTGTTACCA	Cy5	0.1	
Achromobacter xylosoxidans	blaOxa-114	F	CACGAGCCGGTCTGGAA		1.875	
		R	GTGAATACCAGACCACCGAATAC	FAM	1.875	
		P	TACCAGCCYGCCTATCCCGACT		2.5	

43 Gadsby NJ, McHugh MP, Russell CD, Mark H, Conway Morris A, Laurenson IF, et al. Development of two real-time multiplex PCR assays for the detection and quantification of eight key bacterial pathogens in lower respiratory tract infections. Clin Microbiol Infect [Internet]. 2015;21(8):e788.e1-788.e13. Available from: http://dx.doi.org/10.1016/j.cmi.2015.05.004

44 Corless CE, Guiver M, Borrow R, Fox a J, Kaczmarski EB. Simultaneous Detection of Neisseria meningitidis, Haemophilus influenzae, and Streptococcus pneumoniae in Suspected Cases of Meningitis and Septicemia Using Real-Time PCR. J Clin Microbiol. 2001;39(4):1553–8

45 Winchell JM, Thurman KA, Mitchell SL, Thacker WL, Fields BS. Evaluation of three real-time PCR assays for detection of Mycoplasma pneumoniae in an outbreak investigation. J Clin Microbiol. 2008;46(9):3116–8

46 Mitchell SL, Budhiraja S, Thurman KA, Lanier Thacker W, Winchell JM. Evaluation of two real-time PCR chemistries for the detection of Chlamydophila pneumoniae in clinical specimens. Mol Cell Probes [Internet]. 2009;23(6):309–11. Available from: http://dx.doi.org/10.1016/j.mcp.2009.07.005

47 Nazarian EJ, Bopp DJ, Saylors A, Limberger RJ, Musser KA. Design and implementation of a protocol for the detection of Legionella in clinical and environmental samples. Diagn Microbiol Infect Dis. 2008;62(2):125–32

48 Barletta F, Vandelannoote K, Collantes J, Evans CA, Arévalo J, Rigouts L. Standardization of a TaqMan-based real-time PCR for the detection of Mycobacterium tuberculosis-complex in human sputum. Am J Trop Med Hyg. 2014;91(4):709–14

49 Pernica JM, Moldovan I, Chan F, Slinger R. Real-time polymerase chain reaction for microbiological diagnosis of parapneumonic effusions in Canadian children. Can J Infect Dis Med Microbiol. 2014;25(3):151–4

Table.S2 Primers and probes for identification of viral pathogens

		Sequence 5'→3'	Probe dye	Concentration [μM]	Reference
Influenza A	F	GAC CRA TCC TGT CAC CTC TGA C		0.8	(50)
	R	AGG GCA TTY TGG ACA AAK CGT CTA		0.8	
	P	TGC AGT CCT CGC TCA CTG GGC ACG	FAM	0.2	
Influenza B	F	TCCTCAACTCACTCTTCGAGCG		0.5	(51)
	R	CGGTGCTCTTGACCAAATTGG		0.5	
	P	CCAATTCGAGCAGCTGAAACTGCGGTG	YY	0.1	
Respiratory syncytial virus	F1	GGCAAATATGGAAACATACGTGAA		0.5	(52)
	F2	TCTTTTTCTAGGACATTGTAYTGAACAG		0.25	
	R	GCACCCATATTGTTAGTGATGCAG			
	P	CTTCACGAAGGCTCCACRTACACAGC	TAMRA	0.1	
Rhinovirus	F1	CY(lnaA)GCC(lnaT)GCGTGGC		0.35	(53)
	F2	CY(lnaA)GCC(lnaT)GCGTGGTGC		0.35	
	R	GAAACACGGACACCCAAAGTA		0.5	
	P	TCCTCCGGCCCCTGAATGYGGC	Cy5	0.2	
Parainfluenza 1	F	GTT GTC AAT GTC TTA ATT CGT ATC AAT AAT T		0.9	(54)
	R	GTA GCC TMC CTT CGG CAC CTA A		0.9	
	P	TAG GCC AAA GAT TGT TGT CGA GAC TAT TCC AA	FAM	0.2	
Parainfluenza 2	F	GCA TTT CCA ATC TTC AGG ACT ATG A		0.9	(54)
	R	ACC TCC TGG TAT AGC AGT GAC TGA AC		0.9	
	P	CCA TTT ACC TAA GTG ATG GAA TCA ATC GCA AA	JOE	0.2	
Parainfluenza 3	F	AGT CAT GTT CTC TAG CAC TCC TAA ATA CA		0.9	(54)
	R	ATT GAG CCA TCA TAA TTG ACA ATA TCA A		0.9	
	P	AAC TCC CAA AGT TGA TGA AAG ATC AGA TTA TGC A	TAMRA	0.2	
Parainfluenza 4	F	CCTGGAGTCCCATCAAAAGT		0.6	(55)
	R	GCATCTATACGAACACCTGCT		0.6	
	P	AATTGTATTGTCTTGATCAACAAATTTTGAGAC	Cy5	0.34	

**Coronavirus OC43**		F	CGA TGA GGC TAT TCC GAC TAG GT		0.5	(56)
		R	CCT TCC TGA GCC TTC AAT ATA GTA ACC		0.4	
		P	TCC GCC TGG CAC GGT ACT CCC T	FAM	0.5	
**Coronavirus NL63**		F	CAG GGC TGA CAA GCC TTC TCA		0.7	(56)
		R	GCA TCA ACA CCA TTC TGA ACA AGA		0.7	
		P	CGT TGG A**G**G CGT GTT CCT ACC AGA GAG G	HEX	0.15	
**Coronavirus 229E**		F	TGG AAG TGC AGG TGT TGT GGC		0.7	(56)
		R	TGA CTA TCA AAC AGC ATA GCA GCT G		0.3	
		P	CCA CAA TTT GCT GAG CTT GTG CCG TC	TAMR A	0.15	
**Coronavirus HKU1**		F	CAC TTC TAT TCC CTC CGA TGT TTC		0.5	(56)
		R	TTA GAA GCA GAC CTT CCT GAG CC		0.3	
		P	CGC CTG GTA CGA TTT TGC CTC AAG GCT	Cy5	0.1	
**Adenovirus**		F 1	GCC GCA GTG GGC **D**TA CA TGC ACA TC		0.2	(57)
		F 2	GCC CCA GTG GGC ATA CA TGC ACA TC		0.5	
		R	GCC AC**S** GTG GGG TT**Y** CTA AAC TT		0.5	
		P	TGC ACC AGA CCC GG**R** CTC AGG TAC TCC GA	TAMR A	0.4	
**Bocavirus**		F	GGA AGA GAC ACT GGC AGA CAA		0.3	(55)
		R	GGG TGT TCC TGA TGA TAT GAG C		0.3	
		P	CTG CGG CTC CTG CTC CTG TGA T	YY	0.15	
**Metapneumovirus**		F	GAA GAR ATA GAC AAA GAR GCA AG		0.25	(55)
		R	TCC CAC TTC TAT KGT TGA TGC TAG		0.1	
		P	TCA GCA CCA GAC ACA CC	Cy5	0.2	
**Human Enterovirus**		F	CCCTGAATGCGGCTAATCC		0.5	(58)
		R	ATTGTCACCATAAGCAGCCA		0.5	
		P	CAGCGAACCGACTACTTTGGG	FAM	0.2	
**SARS-CoV-2**	N 1	F	GAC CCC AAA ATC AGC GAA AT		0.4	(59)
		R	TCT GGT TAC TGC CAG TTG AAT CTG		0.4	
		P	ACC CCG CAT TAC GTT TGG TGG ACC	FAM	0.05	
	N 2	F	GAC CCC AAA ATC AGC GAA AT		0.4	(59)
		R	GCG CGA CAT TCC GAA GAA		0.4	

P	ACA ATT TGC CCC CAG CGC TTC AG	Cy5	0.15

50 Who T, Centre C, Atlanta CDC, States U. CDC protocol of realtime RTPCR for influenza A(H1N1). 2009;1(October).

51 Selvaraju SB, Selvarangan R. Evaluation of three influenza A and B real-time reverse transcription-PCR assays and a new 2009 H1N1 assay for detection of influenza viruses. J Clin Microbiol. 2010;48(11):3870–5.

52 Fry AM, Chittaganpitch M, Baggett HC, Peret TCT, Dare RK, Sawatwong P, et al. The burden of hospitalized lower respiratory tract infection due to respiratory syncytial virus in rural thailand. PLoS One. 2010;5(11).

53 Lu X, Holloway B, Dare RK, Kuypers J, Yagi S, Williams J V., et al. Real-time reverse transcription-PCR assay for comprehensive detection of human rhinoviruses. J Clin Microbiol. 2008;46(2):533–9

54 Watzinger F, Suda M, Preuner S, Baumgartinger R, Ebner K, Baskova L, et al. Real-time quantitative PCR assays for detection and monitoring of pathogenic human viruses in immunosuppressed pediatric patients. J Clin Microbiol. 2004;42(11):5189–98.

55 Templeton KE, Scheltinga SA, Beersma MFC, Kroes ACM, Claas ECJ. PCA Data Template for Ryan (2). 2004;42(4):1564–9.

56 Tiveljung-Lindell A, Rotzén-Ostlund M, Gupta S, Ullstrand R, Grillner L, Zweygberg-Wirgart B, et al. Development and implementation of a molecular diagnostic platform for daily rapid detection of 15 respiratory viruses. J Med Virol. 2009 Jan;81(1):167–75

57 Heim A, Ebnet C, Harste G, Pring-Åkerblom P. Rapid and quantitative detection of human adenovirus DNA by real-time PCR. J Med Virol. 2003;70(2):228–39

58 Tryfonos C, Richter J, Koptides D, Yiangou M, Christodoulou CG. Molecular typing and epidemiology of enteroviruses in Cyprus, 2003-2007. J Med Microbiol. 2011;60(10):1433–40

59 Lu, X., Wang, L., Sakthivel, S. K., Whitaker, B., Murray, J., Kamili, S., Lynch, B., Malapati, L., Burke, S. A., Harcourt, J., Tamin, A., Thornburg, N. J., Villanueva, J. M., & Lindstrom, S. (2020). US CDC real-time reverse transcription PCR panel for detection of severe acute respiratory syndrome Coronavirus 2. *Emerging Infectious Diseases*, *26*(8), 1654–1665. https://doi.org/10.3201/eid2608.201246
